# Bursting at the Seams: Molecular Mechanisms Mediating Astrocyte Swelling

**DOI:** 10.3390/ijms20020330

**Published:** 2019-01-15

**Authors:** Audrey D. Lafrenaye, J. Marc Simard

**Affiliations:** 1 Department of Anatomy and Neurobiology, Virginia Commonwealth University, Richmond, VA 23298, USA; 2 Departments of Neurosurgery, University of Maryland School of Medicine, Baltimore, MD 21201, USA; 3 Departments of Pathology, University of Maryland School of Medicine, Baltimore, MD 21201, USA; 4 Departments of Physiology, University of Maryland School of Medicine, Baltimore, MD 21201, USA

**Keywords:** astrocyte, swelling, gap junction channels, aquaporin, TRPV4, Kir4.1, Na^+^/K^+^-ATPase, NKCC, SUR1-TRPM4, VRAC

## Abstract

Brain swelling is one of the most robust predictors of outcome following brain injury, including ischemic, traumatic, hemorrhagic, metabolic or other injury. Depending on the specific type of insult, brain swelling can arise from the combined space-occupying effects of extravasated blood, extracellular edema fluid, cellular swelling, vascular engorgement and hydrocephalus. Of these, arguably the least well appreciated is cellular swelling. Here, we explore current knowledge regarding swelling of astrocytes, the most abundant cell type in the brain, and the one most likely to contribute to pathological brain swelling. We review the major molecular mechanisms identified to date that contribute to or mitigate astrocyte swelling via ion transport, and we touch upon the implications of astrocyte swelling in health and disease.

## 1. Introduction

Astrocytes, loosely translated as “star cells” in Greek, are named for their stellate appearance. Astrocytes are one of the most well-known glial cell types in the central nervous system (CNS) and serve a multitude of functions including, but not limited to, blood-brain barrier (BBB) regulation, glial scar formation, regulation of synaptic activity, innate inflammatory responses and glucose storage. Astrocytes have been divided into traditional, type-1 astrocytes, and NG2+, type-2 astrocytes, which some groups now think may be a distinct glial population with unique properties different from traditional astrocytes [1]. As many studies of astrocytes have not been able to reliably differentiate these populations, however, both NG2+ glia and traditional astrocytes will be operationally defined as astrocytes for the purposes of this review. Astrocytes associate with each other and with oligodendrocytes through gap junctions to form a panglial syncytium, a complex and wide-reaching network of interconnected glia. A primary function of this syncytium is the redistribution of ions and small osmotically active molecules, primarily K^+^, from regions of elevated concentration to distant regions, where deposition of additional osmolytes won’t impact the microenvironment. This redistribution of ions was coined K^+^ spatial buffering by Orkand and colleagues in 1966, when they hypothesized that astrocytes take up excess K^+^ from the parenchyma and release an equal amount of K^+^ a distance away to prevent local buildup [2]. In the healthy brain, this K^+^ spatial buffering does not result in substantial astrocyte swelling, although it has been shown to result in local astrocyte swelling and correlative shrinkage at distant sites [3,4]. As astrocytes are water permeable, water also travels in this syncytium between the cerebral spinal fluid (CSF) and the parenchyma through astrocyte end-feet that are connected to the periarteriolar Virchow-Robin space, the space immediately inside the blood brain barrier, adjacent to the endothelial cells lining the vasculature [5,6].

Astrocytes react differently to varying amounts of excess extracellular water. When exposed to solutions that are modestly hypoosmotic, astrocytes swell within seconds then quickly return to pre-exposure volume within minutes [7,8]. However, following exposure to more severely hypoosmotic solutions, astrocytes remain swollen and demonstrate oscillations of Ca^2+^ waves that are not observed with less hypoosmotic solutions [8].

**Figure 1:**
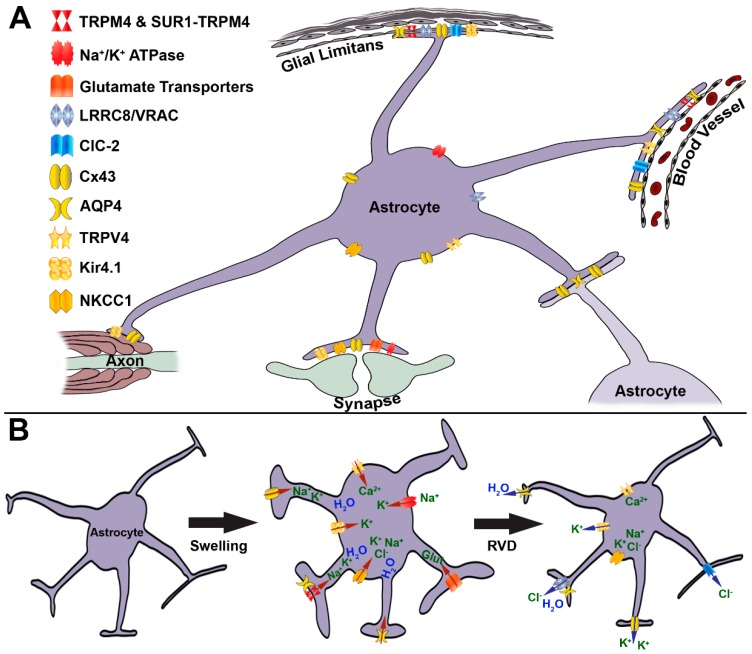
Astrocyte swelling and volume regulation involve multiple complex processes. (**A**) Illustration depicting the major sites associated with movement of osmotically active molecules into and out of astrocytes influencing astrocyte swelling. Astrocytic end feet at both the glial limitans and surrounding parenchymal blood vessels have been well documented to play a role in astrocyte volume change via movement of osmolytes and water. Molecules also move through the panglial syncytium through contacts with both oligodendrocytes at the paranode and with neighboring astrocytes. Uptake of osmolytes at synapses is also involved in alterations in astrocyte volume. The potential localization of channels reported to regulate astrocyte swelling and/or volume decrease are also depicted; however, exact subcellular localization for many of these channels remains to be determined. (**B**) Summary of channels mediating astrocyte swelling (red channels) or regulated volume decrease (RVD; blue channels). Yellow channels are involved in both swelling and RVD. During astrocyte swelling, the channels colored red are involved in mediating the influx of ions and osmotically active molecules (red arrows). Swelling involves K^+^ ions moving into the cell via Cx43 gap junctions and hemichannels, Kir4.1 and Na^+^/K^+^-ATPase. The NKCC1 and SUR1-TRPM4 channels allow the influx of multiple ions, including K^+^, Na^+^ and Cl^−^. Glutamate movement into astrocytes through transporters and Ca^2+^ influx through TRPV4 channels also increases the osmotic gradient leading to water movement into swelling astrocytes through AQP4 channels. Following swelling, the channels colored blue are involved in reducing astrocytic volume and expelling osmolytes (blue arrows). Upon RVD, K^+^ moves out of individual astrocytes via Cx43, and Kir4.1 channels. Both ClC-2 and LRRC8/VRAC channels remove Cl^−^ from astrocytes resulting in water movement out of astrocytes through AQP4 channels. The TRPV4 and NKCC1 channels also might play roles in mediating astrocyte volume decrease, however, the mechanisms by which this happens are not yet understood. It is important to note that astrocyte swelling and RVD are complex processes with multiple players that may or may not act together in any given situation and/or following any particular pathological event and that our knowledge regarding many of these mechanisms is still limited, therefore parts of this figure are speculative.

Many molecules have been shown to play important roles in mediating astrocyte swelling (Figure 1A). However, not all of these molecules are active in every astrocyte under all swelling conditions. Astrocyte swelling and volume regulation are complex processes with multiple players that may or may not act together in any given situation and/or following any particular pathological event. Additionally, while astrocytes are ubiquitously distributed throughout the CNS, they are not homogeneous. Rather, astrocytes are highly variable, even within the same tissue [9,10]. Astrocytes differ in their ability to take up and sequester glutamate, in their electrophysiological properties, including their resting membrane potentials, and in the extent to which they are connected to the panglial syncytium through gap junction expression [9,11,12]. Astrocytes also differ in terms of their swelling dynamics between rodent strains and even between subpopulations within the same mouse cortex [13,14,15,16]. Below, we discuss some of the molecular mechanisms involved in inducing and transducing astrocyte size alterations, both swelling and shrinkage.

## 2. Techniques for Assessing Astrocyte Swelling

One of the major difficulties in assessing astrocyte swelling in vivo is the fact that the primary markers for astrocyte identity, glial fibrillary acidic protein (GFAP) and vimentin, are intermediate filament proteins. As such, these labels only permit the visualization of a fraction of the total astrocyte cell and process arborization [17]. To overcome this limitation, many researchers investigating the molecular mechanisms of astrocyte swelling utilize in vitro approaches, in which primary rodent astrocytes are cultured, allowing for easier assessment of cell volume changes under various conditions.

### 2.1. In vitro Studies

In culture, astrocytes are usually plated in a monolayer, allowing for the assessment of cell area using differential interference contrast microscopy, in which the difference in the optical path of media as compared to adherent cells is used to create a “shadow” outlining the cell area for easy analysis [18,19]. Similarly, phase contrast microscopy, in which high-contrast images are produced that allow for the assessment of transparent cell boarders, has also been utilized to assess astrocyte volume changes [20]. Lisjak et al. used high-resolution atomic force microscopy of cultured astrocytes to “feel” the borders of the cells prior to and following alterations in the osmolality of the culture medium [21]. They also utilized fluorescent sulforhodamine 101 (SR101) treatment to label astrocytes prior to fixation, allowing them to assess the astrocyte somal area as well [21].

Groups have also used fluorescent calcein AM to both measure cell area and to assess water movement. In this technique, cultured astrocytes are pre-loaded with fluorescent calcein-AM, which diffuses into healthy cells [22,23]. This fluorescent signal can be visualized using live-cell fluorescent microscopy to identify the cytoplasmic area of cultured cells, and therefore assess cell swelling in various scenarios [22,23]. Proteins and salts quench calcein-AM; therefore, dilution of intracellular molecules by water influx is reflected by an increase of the fluorescence intensity of intracellular calcein-AM. This intensity change can be used as a measure of cellular water influx into astrocytes [8,20,22,23,24]. Similarly, the 3-*O*-methyl-[^3^H]-glucose (OMG) equilibration method assesses the radioactivity of astrocytes pre-treated with OMG. The radioactivity of astrocyte lysates reflects the amount of intracellular water when normalized to both the radioactivity of the OMG-containing media as well as the protein concentration of the lysate [25,26,27].

Electrophysiologists have utilized basic properties of system conductance to assess surrogate properties of swollen astrocytes. Specifically, as cells within a fixed volume chamber increase in size, the resistance of the chamber increases proportionally. This readout has been used to approximate astrocyte volume changes in various situations in vitro [28,29]. This method was also used to demonstrate that astrocyte swelling is associated with the unfolding of the plasma membrane rather than its expansion by exocytosis [30].

Notably, numerous in vitro studies utilize the basic principles of water movement by adding hypotonic solution to their astrocyte cultures and simply assume cellular swelling without directly measuring it [31,32].

### 2.2. In vivo and In Situ Histological Studies

While studying astrocyte swelling in situ is difficult, due to the cytoskeletal localization of GFAP and vimentin, various groups have devised creative methods for astrocyte volume assessments in CNS tissue. The advent of transgenic mice, and the attendant ability to generate various fluorescent reporter lines, including those specific to astrocytes, has made in situ astrocyte swelling assessments more feasible. Using either the Cx43-eGFP or the GFAP-eGFP transgenic mouse lines, in which a green fluorescent molecule is expressed only in the astrocyte population, groups have been able to use confocal microscopy to obtain 3D reconstructions of resident tissue astrocytes to assess single cell volume in situ [33,34,35].

One group used intravenous infusion of the fluorescent astrocyte marker, SR101, paired with two-photon microscopy through a cranial window to assess astrocyte soma area, and therefore swelling changes in vivo following water injections [20]. This approach allowed for the assessment of astrocyte soma area in the living brain following hyponatremia. Other groups have looked at astrocyte swelling dynamics in living CNS tissue using hippocampal slice cultures. Lee et al. evaluated the optical signal prior to and after electrical stimulation to measure section thickness, which would indicate changes in cell volume within the tissue under different conditions [36]. Another group used tetramethyl ammonium (TMA^+^)-sensitive microelectrodes to assess the size of the extracellular space in hippocampal slice cultures under different conditions. As astrocyte swelling induces a reduction in the size of the extracellular space, this serves as an inverse surrogate assessment of astrocyte swelling [37].

**Figure 2:**
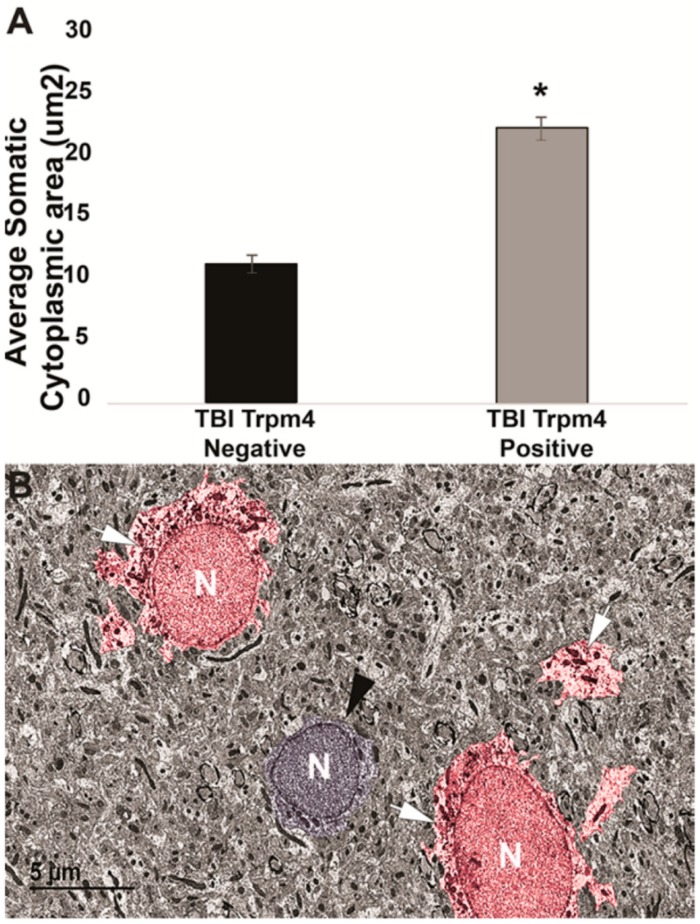
TRPM4 mediates astrocyte swelling after diffuse TBI. (**A**) Bar graph depicting the average area of the somatic cytoplasm surrounding the nucleus. TRPM4 expression nearly doubled the astrocyte cytoplasmic area, compared with TRPM4 -negative astrocytes. (**B**) Representative electron micrograph of astrocytes from the hippocampal gray matter 4 weeks post–central fluid percussion injury labeled against TRPM4 (white arrows; red pseudo color). The arrows indicate immunoreactivity against TRPM4. The astrocyte in the middle of the electron micrograph (black arrow head; blue pseudo color) is a TRPM4-negative astrocyte located between two TRPM4-positive astrocytes. N indicates the nucleus of each cell. Traumatic brain injury (TBI), three animals TRPM4-negative, *n* = 57 cells; TRPM4-positive, *n* = 110 cells. Analysis of variance; error bars represent standard error of the mean. * *p* < 0.05. Scale bar 5 µm. From [38].

The Lafrenaye group used electron microscopy, which allows for the visualization of ultrastructural/subcellular components of CNS tissue, including the plasma membrane [38]. This group used the known ultrastructural characteristics of astrocytes, primarily their euchromatic nuclei and electron lucent cytoplasm, to identify astrocytes and assess the somal area of individual cells with different channel expression profiles (Figure 2) [38].

**Figure 3:**
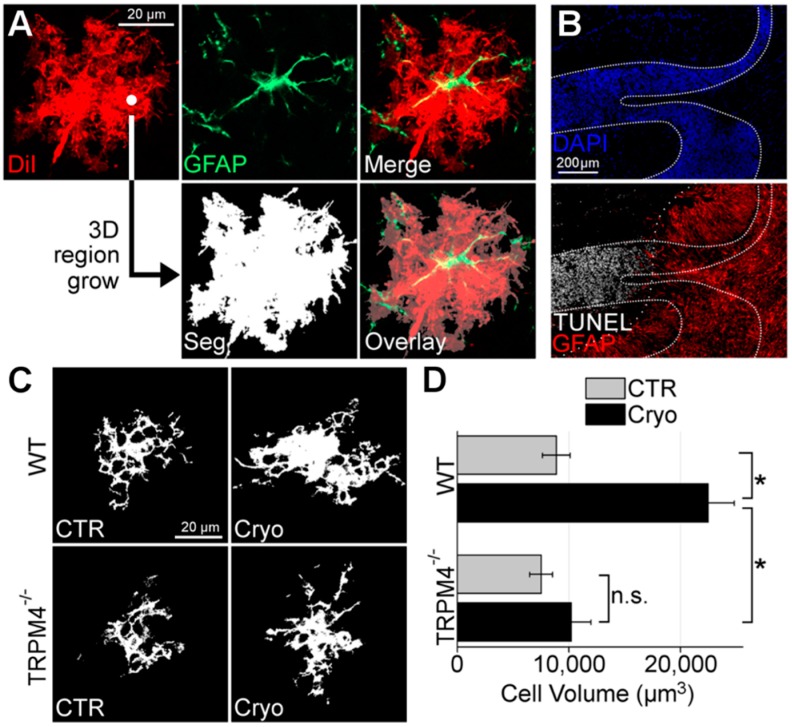
TRPM4 mediates astrocyte swelling after cerebellar cold injury. (**A**) Image processing pipeline for astrocyte volume quantification; DiI-stained (red), GFAP-positive (green) astrocytes were imaged; 3D region growing of DiI image outputs a binary image segmentation (Seg) of intracellular voxels (white) from extracellular voxels (black); overlay image of segmentation with DiI/GFAP image demonstrates full coverage of astrocyte arborization (**B**) Montage of micrographs of murine cerebellum with granule cell layer defined with DAPI (dense dotted lines) and co-labeled for GFAP (red) and TUNEL (white) showing TUNEL-positive granule cell layer tissues do not overlap (sparse dotted line) with GFAP-positive granule cell layer tissues; the results shown are representative of *n* = 4 mice. (**C**) Slices of segmented binary images of cerebellar granule cell layer astrocytes from wild-type (WT) and TRPM4^−/−^ mice submitted to control sham surgery (CTR) or cerebellar cold injury (Cryo) showing that in WT mice, granule cell layer astrocytes in cold injured cerebellum exhibited swelling of somata and processes, whereas astrocytes from TRPM4^−/−^ mice were protected from astrocyte swelling after cerebellar cold injury; the results shown are representative of *n* > 15 cells from 3 independent mice. (**D**) Quantification of granule cell layer astrocytic volume in WT and TRPM4^−/−^ mice submitted to sham surgery (CTR) or cerebellar cold injury (Cryo) showing that after cerebellar cold injury, WT astrocytes increased in volume from 8.86 × 10^4^ μm^3^ to 22.47 × 10^4^ μm^3^; TRPM4^−/−^ astrocytes increased in volume from 7.5 × 10^4^ μm^3^ to only 10.2 × 10^4^ μm^3^; TRPM4 knockout led to significant reduction in astrocyte swelling after cold injury; * *p* < 0.05 in ANOVA with Tukey tests between groups denoted with brackets; n.s. = non-significant; *n* > 15 cells from 3 different mice. From [23].

Researchers who aimed to assess astrocyte swelling in fixed CNS tissue have devised innovative solutions. Sullivan et al. paired GFAP immunohistochemistry with traditional Golgi staining to assess the total area of astrocytes following hypoxia/ischemia in neonatal pigs [39]. Golgi staining labels the entire plasmalemma of a small population of random CNS cells, including neurons and glia, so the incorporation of both Golgi stain and GFAP labeling allowed the group to assess individual astrocyte areas. Using the same principle of diffuse random labeling of CNS cells, the Simard group used a gene gun to deliver diolistic labeling to fixed tissue sections [23]. They paired this method with GFAP labeling to allow for the identification, imaging of 3D confocal reconstructions, and specific analysis of astrocyte cell volume within the CNS tissue (Figure 3) [23].

## 3. Implication of Astrocyte Swelling in Disease

Pathological astrocyte swelling is associated with a variety of negative consequences. In ischemia, it is proposed that an influx of ions into astrocytes induces swelling and generates an osmotic gradient between the brain and the blood that drives ion movement from the blood into the brain parenchyma, a phenomenon termed ionic edema [40]. Excess movement of ions and water into brain cells leads to extreme cellular swelling, which could result in oncotic cell death of astrocytes and other CNS cells [41].

Astrocyte swelling inherently reduces the size of the extracellular space [42]. The reduction in extracellular space may elevate extracellular ion concentrations, which raises neuronal resting membrane potentials, and can affect neuronal excitability, making neurons more likely to fire in response to any given stimulus [43]. The elevation in resting membrane potentials also may induce epileptiform activity. It has been shown that epileptiform activity can be induced with hypoosmotic solution and can be abolished by increasing extracellular osmolality [42,44,45,46,47,48].

The increase in synchronous neuronal activity can precipitate increases in excitatory neurotransmitter release and result in higher localized concentrations of excitatory amino acids (EAA) such as glutamate. Healthy astrocytes remove extraneous EAAs from the extracellular environment. Swollen astrocytes, however, do not take up EAAs. Instead, upon swelling, astrocytes release EAAs, including glutamate, into the extracellular environment [31]. Release of EAAs from swollen astrocytes is inversely proportional to the extracellular osmolality so that, as the extracellular osmolality decreases, the release of EAAs from swollen astrocytes increases [49,50]. The increase in extracellular EAAs can result in overactivation of neurons leading to excitotoxicity.

Additionally, astrocyte swelling represents a major component of brain swelling in TBI, in which brain tissue retains more water than normal [51]. The cranial vault is a closed, ridged container in which brain tissue, blood and CSF all reside. As astrocytes swell, the pressure within the cranium, the intracranial pressure, increases, and the end feet of swollen astrocytes may compress capillaries [43,52]. Therefore, astrocyte swelling and attendant increases in intracranial pressure can precipitate global ischemia, if the blood pressure is not sufficient to overcome the pressure within the cranium.

## 4. Molecular Mechanisms of Astrocyte Swelling

### 4.1. Gap Junction Channels

As mentioned above, there is a panglial syncytium consisting of astrocytes and oligodendrocytes that are interconnected by gap junctions, which allow for relatively unrestricted movement of small molecules within and between glial cells [53]. This system allows for the maintenance of the K^+^ gradient, in which there is more K^+^ intracellularly than extracellularly. Following neuronal action potential firing, K^+^ is released from the axon by Kv1.1 channels at the juxtaparanode into the paranodal space between the axolemma and the first wrap of myelin [37,54]. This increase in extracellular K^+^ negatively impacts repolarization and raises the neuronal membrane resting potential, making subsequent firing more likely. This K^+^ is moved through the subsequent wraps of myelin via gap junctions [54]. Astrocyte processes are also connected to the outer aspect of the oligodedrocytic paranodal loops through gap junctions [54]. This intimate oligodendrocyte/astrocyte connection at the paranode allows astrocytes to take up extra K^+^ passing through the myelin via gap junctions of the panglial syncytium (Figure 1).

Astrocytes express three main subunits of gap junctional (intercellular communication) and hemichannel (extracellular communication) proteins, connexin26 (Cx26), connexin30 (Cx30) and connexin43 (Cx43), although Cx43 is thought to be most involved in astrocyte-astrocyte intercellular communication [55,56]. Blockade of hemichannels, but not gap junctional connexins, demonstrated benefits following edema-inducing injuries, including reductions in total tissue swelling, diminished neuronal loss and decreased lesion volume [57,58]. Inhibition or loss of both hemichannel and gap junctional Cx43 in cultured astrocytes resulted in reduced astrocyte swelling [8,24]. Knocking out both Cx30 and Cx43 in astrocytes in vivo resulted in swelling of the astrocytic end feet around blood vessels, increased tissue vacuolization, caused higher astrocyte activation and disrupted the blood brain barrier [59,60]. Gap junctional Cx43 has been demonstrated to be disrupted in injuries that typically involve astrocyte swelling and edema, such as traumatic brain and spinal cord injuries [61]. Double knock-out of Cx30/Cx43 in astrocytes following spinal cord injury reduced astrocyte activation and enhanced electrophysiological and behavioral recovery, indicating that in edema-causing injury, Cx43 may be more harmful than helpful [62].

Whether any of the effects of knocking out total astrocytic Cx43 are primarily due to the inhibition of the hemichannel or gap junction functions is not clear. It is, however, known that not all astrocytes express Cx43 gap junctional proteins. A subset of GluR astrocytes do not express Cx43 and have no gap junctional coupling with neighboring astrocytes [63]. These GluR astrocytes have unique morphologies and electrophysiological properties compared to Cx43 expressing GluT astrocytes, and do not appear to be regionally segregated [63]. Rather, GluR astrocytes are interspersed with GluT astrocytes throughout the hippocampus [63,64].

### 4.2. AQP4

Aquaporins (AQP) are non-selective, bidirectional water channels that allow for the passive diffusion of water across cellular membranes due to osmotic gradients. There are three AQPs expressed in the CNS, AQP1, AQP4 and AQP9 [65,66,67,68]. Human astrocytes within the grey matter express AQP4, while those in the white matter express AQP1 [69]. Rodent astrocytes do not express AQP1, but do express both AQP4 and AQP9, with AQP4 being specifically localized to the astrocytic end feet, which are in direct apposition to blood vessels of the CNS (Figure 1) [5,69,70]. AQP9 has been linked to astrogliosis, since its expression is associated with changes in astrocyte morphology, mainly with a higher number of processes [71]. There are six isoforms of AQP4, a-f; however, only AQP4c and AQP4e demonstrated water permeability in cultured oocytes [72].

Increases in AQP4 expression positively correlate with increases in astrocyte volume over time following induction of astrocyte swelling [27]. Hypotonicity and hypothermia have been shown to increase AQP4 expression and localization on the astrocyte membrane, with hypothermia-meditated, but not hypotonicity-mediated, expression requiring activity of the cation channel, transient receptor potential vanilloid 4 (TRPV4) [73].

The localization of AQP4 at the membrane of astrocytes in various regions, including the end feet surrounding the vasculature, is maintained by agrin, laminin, and α-syntrophin [5,42,74]. Mislocalization of AQP4 due to loss of α-syntrophin can precipitate reduction in astrocyte swelling in the face of hypotonic solution, but also can generate inappropriate responses to intracellular K^+^ [34,42]. Loss of AQP4 also has varied effects. In vitro AQP4^−/−^ astrocytes demonstrate slower swelling and recovery kinetics and smaller, slower or absent Ca^2+^ waves following the administration of hypotonic solution [21,22,75]. Overexpression of AQP4 results in faster swelling and recovery dynamics, but reduced changes in astrocyte volume overall, demonstrating a likely role for AQP4 in mediating dynamic changes in astrocyte volume [21]. Another study found that astrocyte swelling was only reduced following priming by certain hypoosmotic concentrations [42,75]. Astrocytes lacking AQP4 are also connected to a greater degree by gap junctions and are more efficient at K+ spatial buffering than their AQP4 expressing counterparts [42,76]. Edema-inducing traumatic brain injury in AQP4^−/−^ mice demonstrated less extracellular K^+^, more efficient K^+^ spatial buffering, reduction in blood brain barrier breakdown and behavioral improvements [77]. In another study, however, absence of AQP4 resulted in an increase in activity-induced tissue swelling [78].

AQP4 is involved in more than water movement and astrocyte swelling. Downregulation of AQP4 or AQP4’s stabilizing proteins, α-syntrophin or dystroglycan alters the phosphorylation of focal adhesion kinase and reduces astrocytic process outgrowth, demonstrating a role for AQP4 in cytoskeletal dynamics as well [79].

After CNS injury, AQP4 shifts from its normal polarized location at the astrocyte end feet to widespread expression throughout the plasmalemma [23,80,81]. This phenomenon, termed AQP4 dysregulation, is accompanied by a switch in AQP4 binding partners from TRPV4 to transient receptor potential melastatin 4 (TRPM4). Recently, Stokum et al. demonstrated a physical and functional association between AQP4 and the Ca^2+^-sensitive non-selective cation channel, TRPM4 [23]. The association of TRPM4 and its heterodimerizing partner, SUR1, with AQP4 significantly increased Ca^2+^-induced astrocyte swelling both in vitro and in vivo [23].

### 4.3. TRPV4

Exposure of astrocytes to hypoosmotic stress induced either in culture or by in vivo intraperitoneal injection of water causes a drastic increase in intracellular Ca^2+^ within minutes [75,82]. In culture, astrocytes subjected to hypoosmotic shock demonstrate an approximately seven-fold increase in Ca^2+^ within seconds that returns to baseline within minutes thereafter [82]. In vivo elevations in astrocytic Ca^2+^, specifically within the soma and astrocyte end feet, appear within the first 15 min of intraperitoneal injection of water and continue to increase over the first hour post- injection [75].

TRPV4 is a Ca^2+^ permeable member of the transient receptor potential (TRP) channel family. Activation of TRPV4 leads to drastic increases in intracellular Ca^2+^ that do not occur without TRPV4 [83,84,85,86,87,88]. TRPV4 is expressed in various tissues throughout the body, particularly in the kidney, where it is a key regulator of blood osmolality [89]. In the CNS, TRPV4 is most highly expressed on the membrane of astrocytes, specifically within astrocyte end feet (Figure 1A) [85]. Expression of TRPV4 increases following CNS injuries that are associated with edema and astrocyte swelling [87,88]. The TRPV4 channel is sensitive to multiple physical stimuli, including temperature and pressure/cell swelling [90,91].

These activation mechanisms are specific and uniquely linked to their mechanisms of activation. Temperature-induced TRPV4 activation is mediated through phosphorylation of its tyrosine-555 (tyr-555). However, mechanical cellular strain, such as that which occurs with astrocyte swelling, is unaffected by mutations of tyr-555. Rather, swelling-induced activation of TRPV4 may be mediated through phosphorylation of tyrosine-253 (tyr-253) [92,93]. One study found that TRPV4 associates with src-kinases via the SH2 domain, having the highest affinity for lyn kinase, and mutations of TRPV4 at tyr-253 abolished swelling-induced TRPV4 activation [93]. However, a subsequent study found no change in TRPV4 activation with a mutation of tyr-253 [92]. Rather, they found that Ca^2+^ signaling induced by swelling-mediated TRPV4 activation required breakdown of intracellular arachidonic acid, potentially by phospholipase A2, into epoxyeicosatrienoic acids [92,94]. These divergent findings highlight potential variability and potential redundancy in swelling-induced TRPV4 activation.

Multiple studies have demonstrated the association between TRPV4 and AQP4 in mediating swelling-induced Ca^2+^ influx (Figure 1B). Inhibition or reduction of AQP4 abolished the influx of Ca^2+^ and swelling in the face of hypoosmotic shock [22,75,95]. Swelling -induced Ca^2+^ influx was reduced with TRPV4 inhibition, regardless of AQP4 expression, indicating that TRPV4 activation, and its attendant Ca^2+^ influx, may be downstream of AQP4 [22].

### 4.4. Kir4.1

Astrocyte membranes are highly permeable to K^+^ ions, leading to a hyperpolarized resting membrane potential and low input membrane resistance. The main player in mediating these properties is the Kir4.1 inward rectifying K^+^ channel [42,96]. Without Kir4.1, astrocytes lack their signature K^+^ currents, which are sensitive to K^+^ blockers such as Ba^2+^ [97,98]. The Kir4.1 channels are expressed throughout the brain, but are found in the highest concentrations in the olfactory bulb, cerebellum, brain stem, spinal cord and midbrain. While Kir4.1 is not expressed on neurons, it is expressed in a variety of CNS glia, including oligodendrocytes, and astrocytes. Protoplasmic astrocytes within the grey matter have higher Kir4.1 expression than fibrous astrocytes within the white matter [96,99]. The expression of Kir4.1 increases with age, particularly within the first 10 days postnatally [33,97]. This increase is associated with both an increase of the inward current in developing astrocytes and a shift away from oligodedroglial expression of Kir4.1 [33,97].

Many groups have shown both physical and functional associations between the water channel, AQP4, and Kir4.1 [42,76]. The passive movement of K^+^ through Kir4.1 channels has been theorized to be particularly important for K^+^ spatial buffering (Figure 1) [42,98,100,101]. However, other groups found that Kir4.1 was not involved in extracellular K^+^ clearance [29,102,103]. Additionally, some groups demonstrated a reduction in Kir4.1 expression that is linked to astrocyte swelling following edema-inducing pathologies or following reduction using siRNAs against Kir4.1 [104,105,106]. However, others found no alterations in astrocyte swelling in the absence of Kir4.1 (Figure 1B) [29]. One group found that while there was no change in astrocyte soma swelling with Kir4.1 reduction or inhibition, astrocyte processes began swelling without Kir4.1, demonstrating a subcellular alteration in terms of swelling properties, which could explain some of the divergent findings [35]. Another possibility is that different subpopulations of astrocytes respond differently to reductions in Kir4.1 based on their swelling properties. High responding astrocytes, which have large volume changes upon induction of swelling, were shown to respond to Kir4.1 inhibition with reductions in overall swelling volume, whereas low responding astrocytes, that do not demonstrate drastic volume changes with swelling-induction by oxygen-glucose deprivation, increased their volume when Kir4.1 was absent [12].

### 4.5. Na^+^/K^+^-ATPase and NKCC

The Na^+^/K^+^-ATPase transporter uses ATP to move Na^+^ and K^+^ against their electrochemical gradients and is expressed by both neurons and glia. This transporter is involved in uptake of extracellular K^+^ by astrocytes, especially within the first few seconds of extracellular K^+^ elevation [29,42,102,103]. The Na^+^/K^+^-ATPase transporter has five different subunits, four of which are expressed by astrocytes (α1, α2, β1 and β2), that determine the transporter’s K^+^ and voltage sensitivity [29]. The α2β1 Na^+^/K^+^-ATPase subunit combination is the most efficient in terms of K^+^ clearance [29]. As K^+^ is an osmotically active ion, movement of K^+^ into the astrocyte through the Na^+^/K^+^-ATPase can result in acute astrocyte swelling.

The other important player in K^+^ clearance from the extracellular space is the Na^+^-K^+^-Cl^−^ cotransporter (NKCC) [29,102,103]. There are two isoforms, NKCC1 and NKCC2, of which NKCC1 is expressed on neurons and glia, including astrocytes [52,107,108,109]. Phosphorylation of NKCC1 induces translocation to the cell membrane where NKCC1 is active [52]. Upon activation, NKCC1 moves one Na^+^, one K^+^ and two Cl^−^ ions into the cell, which maintains the electrochemical gradient while simultaneously enhancing the osmotic gradient (Figure 1). While N^+^/K^+^-ATPase appears to be activated within the first few seconds of exposure to high extracellular K^+^, the NKCC1 cotransporter is upregulated and activated 20–30 s following increases in extracellular K^+^ [29]. Inhibition of NKCC1 also abolished cell swelling of astrocytes in vitro as well as reducing both intracellular Na^+^ and Cl^−^ [18,29,110]. Release of EAAs was reduced with NKCC1 inhibition in vitro [19]. Reduction in extracellular Na^+^ concentration also abolished astrocyte swelling in vitro, which would have disrupted the function of the NKCC1 cotransporter [19]. Inhibition of NKCC1 in Sprague Dawley rats demonstrated a reduction in TBI-induced brain swelling [111]. NKCC1 inhibition, however, did not appear necessary for either K^+^ clearance or astrocyte swelling in Wistar rat hippocampal slices [29]. This difference in findings could, at least in part, be due to the known variability between rat strains in terms of astrocyte swelling dynamics [112]. Additionally, NKCC1 appears to be necessary only in a subpopulation of astrocytes, in which astrocytes demonstrating high swelling responsiveness to oxygen-glucose deprivation are non-responsive to NKCC1 inhibition [12].

### 4.6. SUR1-TRPM4

Unlike all other molecular mediators mentioned thus far, sulfonylurea receptor 1—transient receptor potential melastatin 4 (SUR1-TRPM4) channels are not normally expressed by astrocytes but are upregulated de novo following ischemic and traumatic CNS injury [113,114,115,116]. The expression of this channel after injury coincides with the time when astrocyte swelling predominates. TRPM4, the pore-forming subunit, is a nonselective monovalent cation channel activated by intracellular Ca^2+^ [117]. SUR1, an ATP-binding cassette transporter that regulates pore-forming subunits, physically co-associates with TRPM4 and doubles its Ca^2+^ sensitivity [113,114,118]. TRPM4 alone can form functional plasmalemmal ion channels in the absence of SUR1, whereas SUR1 does not traffic to the membrane without a pore-forming TRPM4 subunit [119,120].

SUR1-TRPM4 has been implicated in astrocyte swelling [113,114], brain edema and brain swelling (Figure 1) [115]. Recent work focusing on TRPM4 following diffuse, fluid percussion TBI reported that TRPM4-positive astrocytes show significant increases in soma size, assessed using electron microscopy, that correlate with TRPM4 expression [38]. Astrocyte size correlated significantly with TRPM4 intensity/expression in individual cells in the hippocampus. TRPM4-positive astrocytes were found to have nearly double the average soma size, compared with TRPM4-negative astrocytes, consistent with TRPM4 expression being associated with significant swelling of astrocytes post-TBI (Figure 2) [38].

Independent evidence for a role of TRPM4 in astrocyte swelling came from experiments with cold injury, a model that in many ways resembles ischemic injury. Using diolistic labeling, Stokum et al. showed that the volume of astrocytes in the cerebellar granule cell layer increases approximately two-fold at three days after injury in wild-type animals [23]. By contrast, genetic ablation of TRPM4 in TRPM4^−/−^ mice prevented astrocyte swelling (Figure 3), consistent with a key role for TRPM4, and by extension, SUR1-TRPM4, in astrocyte swelling in vivo [23]. The ~two-fold magnitude of change observed by both Gorse et al. and by Stokum et al., albeit referring to areas and volumes, respectively, point to exceptionally large contributions by TRPM4 to astrocyte swelling under pathological conditions.

Notably, a recent clinical trial of large hemispheric infarction that studied pharmacological blockade of SUR1-TRPM4 using intravenous glibenclamide reported that brain swelling, measured as midline shift, was reduced by half with drug [121], giving further evidence of a major role for SUR1-TRPM4 in brain swelling.

## 5. Regulatory Volume Decrease and Volume Regulated Anion Channels

Astrocytes in vitro undergo a regulatory volume decrease (RVD) within minutes of initial swelling [28,42,122,123,124,125]. This phenomenon is theorized to involve movement of anions and other osmotically active molecules, such as EAAs, out of the astrocyte [126]. Broad spectrum anion channel blockers reduce both the Cl^−^ currents and efflux of amino acids following hypoosmotic shock [32]. To maintain electroneutrality, K^+^ cations are thought to move out of the cell, with water following due to the new osmotic gradient. The chloride channel ClC-2, one of few anionic inward rectifier channels, is a potential candidate for regulating astrocyte RVD. ClC-2 is activated by both cell hyperpolarization and exposure to changes in cell volume following hypoosmotic stimulation [127,128]. ClC-2 channels are ubiquitously expressed, including on astrocytes, particularly at the astrocytic end feet (Figure 1) [129,130,131,132]. ClC-2 has also been shown to physically associate with AQP4. Specifically, expression of ClC-2 channels and the osmotically-sensitive Cl^−^ current were reduced with siRNA against AQP4 [133]. Overexpression of the isoform AQP4e significantly enhanced RVD in cultured astrocytes following exposure to hypoosmotic conditions [21]. However, ClC-2 inhibition in astrocyte cultures did not suppress RVD-associated amino acid release or Cl− current [32]. As specific assessments of ClC-2 expression and activity on cell volume changes have not been directly performed, this candidate remains speculative.

Volume regulated anion channels (VRACs) have been shown to open in response to cell swelling, allowing the efflux of Cl^−^ and amino acids which induce RVD. However, the identity of these VRACs has been elusive. Regulation of cell volume following hypoosmotic-induced astrocyte swelling in vitro was demonstrated to require extracellular Ca^2+^, indicating a potential role for Ca^2+^ movement, potentially through the TRPV4 channel (Figure 1B) [28]. However, another group observed functional RVD with either TRPV4 inhibition or in a Ca^2+^-free environment in vitro, arguing against a role for TRPV4 in astrocyte RVD [22]. The NKCC1 transporter might also play a role in regulating RVD, in that RVD dynamics were faster in NKCC1^−/−^ astrocytes in vitro following hypoosmotic-induced swelling compared to astrocytes that expressed NKCC1 (Figure 1B) [18]. Sodium fluoride-induced volume decrease in the hippocampus, however, did not require NKCC1, but did depend on MAPK activation [36]. There is also a possible link between RVD and expression of the intermediate filament proteins, GFAP and vimentin; astrocytes null for both GFAP and vimentin had drastic decreases in osmotic molecule release compared even to single intermediate filament null astrocytes [126].

Recently, the elusive VRACs were identified as members of the leucine-rich repeat containing protein 8 family (LRRC8A-E) [42,134,135]. The LRRC8A channel appears to be required for VRAC-mediated currents and the LRRC8B-E family members are involved in regulation of LRRC8A kinetics [42,136]. LRRC8A is expressed on astrocytes in culture, particularly at astrocytic end feet, and can sense intracellular ionic changes induced with astrocyte swelling, leading to LRRC8A opening (Figure 1) [136,137].

## 6. Two Molecules That Induce Astrocyte Swelling—Glutamate and Ammonia

Glutamate is an EAA neurotransmitter that is osmotically active. It is generated from glutamine, which is synthesized in astrocytes, released into the extracellular space and is taken up by neurons. In the neuron, mitochondrial phosphate-activated glutaminase reacts with glutamine forming glutamate and ammonia [43]. Excess extracellular glutamate is neurotoxic, and so astrocytes take up extracellular glutamate following axonal firing. This glutamate uptake is associated with Na^+^ and water influx into the astrocytes and resultant swelling [138,139,140]. Glutamate is also directly harmful to astrocytes, as astrocyte viability decreases with high levels of extracellular glutamate [13]. Astrocyte soma size, indicative of swelling, was found to be increased in proportion to the amount of extracellular glutamate added to the media in primary astrocyte cultures (Figure 1), possibly through the activation of NKCC1 or Na^+^/K^+^-ATPase in astrocytes, transporters involved in increased astrocyte swelling [12,138]. The Ca^2+^-permeable channel, TRPV4 also may play a role in glutamate release, as TRPV4^−/−^ cells demonstrate a decrease in intracellular glutamate [35].

Liver failure has been associated with reductions in Kir4.1 expression and K^+^ clearance from the extracellular space [105]. Liver failure also results in an increase in circulating ammonia. Ammonia-exposed endothelial cells upregulate NF-κB and reactive oxygen species that lead to astrocyte swelling [25]. If NF-κB is inhibited in endothelial cells exposed to ammonia, or these cells are treated with antioxidants, then astrocyte swelling does not occur [25]. Inhibition of NF-κB also reduced overall brain edema following brain injury [26]. Parenchymal ammonia or lactic acid also leads to astrocyte swelling, potentially by activating acid-sensing channels, which are permeable to Na^+^ and are closed at physiological pH, and/or via ammonia-induced upregulation and activation of NKCC1 [52,110,141,142]. Ammonia, however, also can be taken up by astrocytes for use in making glutamine, leading to oxidative stress and opening of the mitochondrial permeability transition pore (MPT), which increases intracellular ion concentration and astrocyte swelling [110]. If the MPT is inhibited with cyclosporine A, ammonia no longer produces astrocyte swelling in vitro [43]. Inhibition of nitric oxide synthase (NOS) or addition of antioxidants also reduces astrocyte swelling, both in culture and in CNS tissue slices [43,52]. More recently, Jayakumar et al. showed that ammonia-exposed astrocytes upregulate NF-κB that leads to de novo expression of SUR1-TRPM4 channels previously implicated in astrocyte swelling, and that treatment with glibenclamide to inhibit SUR1-TRPM4 significantly reduced brain edema in a rat model of acute liver failure [143].

## 7. Conclusions

Under numerous pathological conditions, including ischemic, traumatic, hemorrhagic and metabolic brain injury, swelling of astrocytes likely contributes in a major way to brain swelling, one of the most robust predictors of patient outcome. Yet, despite its apparent importance, the specific contribution of astrocyte swelling to the overall phenomenon of brain swelling remains elusive, almost impossible to quantify vis-à-vis contributions from other space-occupying mechanisms such as extravasated blood, extracellular edema fluid, vascular engorgement and hydrocephalus. Only by quantifying its relative contribution can we adequately interrogate this mechanism in various CNS diseases, and only by understanding its underlying molecular mechanisms can we actually target astrocyte swelling with the aim of improving patient outcome. At present, our knowledge is wanting, and no therapies exist to address this. Progress is inevitable, however, as is the day when astrocyte swelling will be sufficiently well understood to permit its pharmacological regulation following brain injury.
